# Economic instruments for obesity prevention: results of a scoping review and modified delphi survey

**DOI:** 10.1186/1479-5868-8-109

**Published:** 2011-10-06

**Authors:** Guy EJ Faulkner, Paul Grootendorst, Van Hai Nguyen, Tatiana Andreyeva, Kelly Arbour-Nicitopoulos, M Christopher Auld, Sean B Cash, John Cawley, Peter Donnelly, Adam Drewnowski, Laurette Dubé, Roberta Ferrence, Ian Janssen, Jeffrey LaFrance, Darius Lakdawalla, Rena Mendelsen, Lisa M Powell, W Bruce Traill, Frank Windmeijer

**Affiliations:** 1 Faculty of Physical Education and Health, University of Toronto, 55 Harbord Street, Toronto ON, M5S 2W6, Canada; 2 Leslie L. Dan Faculty of Pharmacy, University of Toronto, 144 College Street, Toronto ON, M5S 3M2, Canada; 3 Rudd Center for Food Policy & Obesity, Yale University, Department of Psychology, 309 Edwards Street, New Haven CT, 06520-8369, USA; 4 Department of Kinesiology, McMaster University, 1280 Main Street West, Hamilton ON, L8S 4K1, Canada; 5 Department of Economics, University of Victoria, PO Box 1700 STN CSC, Victoria, BC, V8W 2Y2, Canada; 6 Friedman School of Nutrition Science and Policy, Tufts University, 150 Harrison Avenue, Boston MA, 02111, USA; 7 Departments of Policy Analysis and Management, and Economics, Cornell University, 3M24 MVR Hall, Ithaca NY, 14853, USA; 8 Center for Public Health Nutrition, School of Public Health, University of Washington, Seattle, WA 98195-3410, USA; 9 Desautels Faculty of Management, McGill University, 1001 Sherbrooke St West, Montreal QC, H3A 1G5, Canada; 10 Ontario Tobacco Research Unit, Dalla Lana School of Public Health, University of Toronto, 33 Russell St., Toronto ON, M5S 2S1, Canada; 11 School of Kinesiology and Health Studies Queen's University, 28 Division St. Kingston ON, K7L 3N6, Canada; 12 Department of Economics, Monash University, Building H4, Room 47 Sir John Monash Road, Caulfield, Victoria 3145, Australia; 13 Schaeffer Center for Health Policy and Economics, University of Southern California, 3335 S. Figueroa St, Unit A, Los Angeles, CA 90089-7273, USA; 14 Ryerson University, School of Nutrition, 350 Victoria Street, Toronto ON, M5B 2N8, Canada; 15 Institute for Health Research and Policy University of Illinois at Chicago, 1747 W. Roosevelt Road, Chicago, IL 60608, USA; 16 Department of Food Economics and Marketing, University of Reading Whiteknights PO Box 237, Reading RG6 6AR, UK; 17 Department of Economics, University of Bristol, 8 Woodland Road, Bristol BS8 1TN, UK

## Abstract

**Background:**

Comprehensive, multi-level approaches are required to address obesity. One important target for intervention is the economic domain. The purpose of this study was to synthesize existing evidence regarding the impact of economic policies targeting obesity and its causal behaviours (diet, physical activity), and to make specific recommendations for the Canadian context.

**Methods:**

Arksey and O'Malley's (2005) methodological framework for conducting scoping reviews was adopted for this study and this consisted of two phases: 1) a structured literature search and review, and 2) consultation with experts in the research field through a Delphi survey and an in-person expert panel meeting in April 2010.

**Results:**

Two key findings from the scoping review included 1) consistent evidence that weight outcomes are responsive to food and beverage prices. The debate on the use of food taxes and subsidies to address obesity should now shift to how best to address practical issues in designing such policies; and 2) very few studies have examined the impact of economic instruments to promote physical activity and clear policy recommendations cannot be made at this time. Delphi survey findings emphasised the relatively modest impact any specific economic instrument would have on obesity independently. Based on empirical evidence and expert opinion, three recommendations were supported. First, to create and implement an effective health filter to review new and current agricultural polices to reduce the possibility that such policies have a deleterious impact on population rates of obesity. Second, to implement a caloric sweetened beverage tax. Third, to examine how to implement fruit and vegetable subsidies targeted at children and low income households.

**Conclusions:**

In terms of economic interventions, shifting from empirical evidence to policy recommendation remains challenging. Overall, the evidence is not sufficiently strong to provide clear policy direction. Additionally, the nature of the experiments needed to provide definitive evidence supporting certain policy directions is likely to be complex and potentially unfeasible. However, these are not reasons to take no action. It is likely that policies need to be implemented in the face of an incomplete evidence base.

## Background

The causes of overweight and obesity, and the potential solutions to prevent and reduce obesity prevalence are complex. We live in an obesogenic environment that increasingly promotes a high energy intake and sedentary behaviour [1]. No single strategy will solve this health problem. Social-ecological theory emphasizes that physical activity and dietary behaviours are influenced by factors across multiple domains including the individual, social, physical and policy spheres. Accordingly, comprehensive, multi-level approaches are required to address obesity. One important target for intervention is the economic domain.

Standard economic theory hypothesizes that individuals make decisions to make themselves as well off as possible. In other words, individuals attempt to satisfy objectives subject to constraints. Both objectives and constraints are germane to diet and physical activity choices [2]. On the objectives side, economics emphasizes that human welfare depends on multiple factors, and individuals make trade-offs between them. If health were the only goal, then there likely would be little obesity and individuals would spend all of their time and money on health-enhancing activities. Clearly this is not the case. Obesity, then, could be the result of the trade-off that individuals make between health and other desired goods, such as the consumption of calorie-rich food and beverages, in order to maximize self-perceived welfare. While preferences are certainly relevant, they alone cannot explain the dramatic increase in obesity prevalence over the last several decades - it seems unlikely that preferences for calorie-rich food or physical (in)activity have changed so suddenly. What *have *changed are: the allocation of time, budget constraints, and technology. Changes in diet and activity can be interpreted as optimizing responses to these changes. In particular, the total (money plus time) price of consuming calorie dense food and beverages has declined and this has at once reduced the price of calories and increased purchasing power. At the same time, higher wage rates and longer hours spent in sedentary employment have made physical activity more expensive. Standard economic theory predicts that these price changes would rationally lead individuals to increase caloric intake and reduce caloric expenditure. An important implication is that, changing prices of calorie dense, unhealthy foods relative to that of low-energy, healthy foods, or altering the cost of physical activity relative to that of sedentary alternatives may lead to changes in diet and physical activity.

While taxes and subsidies are obvious candidates for a government to use to change relative prices, the basis for the government to intervene on obesity is less so. Early rationale for government intervention in obesity focuses on the negative externality of obesity, which argues that obesity results in large health care costs and these costs are borne collectively, so that obesity imposes financial externalities on those who are not obese. While this argument has some merit, there are limitations. While the individuals who are obese likely incur higher health care costs than those individuals who are not obese at any given age, they also have shorter lifespans [3]. Hence, those individuals who are obese might have lower total *lifetime *healthcare costs than those who are not obese.

Recent justification for government intervention rests upon insights from behavioural economics. This literature suggests that the self-control problem could be grounds for government intervention. These economists think of individuals as having two 'selves': a relatively myopic 'today's' self - which is the one that makes diet and physical activity decisions - and a relatively far-sighted 'future' self, which lives with the health consequences. There is sometimes a conflict between the two selves: Today's self may not adequately take into account future self's welfare and succumb to the temptations of calorie rich foods and sedentary lifestyles. The theory suggests that individuals who recognize this dilemma - 'sophisticates' - will use self-commitment devices (e.g., diets, fitness club memberships) to make today's self account for the consequences of their decisions on their future selves. Excise taxation of unhealthy foods or physical activity subsidies can be thought of as a commitment devise to improve the 'future-selves' welfare of non-sophisticates [4,5].

It is worth noting that taxes and subsidies are not free tools. The public finance literature documents a number of costs associated with the use of these tools. First, consumers in the absence of taxes are maximising their utility. Taxes might distort their choice and break this optimality and thus reduce consumer welfare and create a deadweight loss. How large is this deadweight loss depends on elasticity of supply and demand for a good. Second, there are labor and administrative costs required in implementing the tax or subsidy policies. Third, there may be also budgetary costs, especially in the case of subsidies. For example, policies that reward desired behaviours, such as subsidies to physical activity, will create windfall gains to those who already engage in the desired behaviour. This may make the policy a costly way to change relatively few individuals' behaviour.

Economic theory also informs us on potential challenges in applying the taxes and subsidies to change individuals' behaviours. First, in response to price changes, individuals may substitute lower priced goods for higher priced ones. This is the substitution effect. To illustrate, suppose that government decides to apply a special tax on cola. Individuals might then substitute root beer or other kinds of sodas for cola. If governments tax all sodas, then individuals might switch to sugar added sports drinks. As a result of these potential substitutions, the weight of an individual can remain unchanged. Second, taxes increase the prices, which in turn reduce the purchasing power of one's income. The reductions in purchasing power can affect diet and physical activity choices in a way that can mute the effectiveness of tax policies in controlling weight. For example, if people spend a lot of their budget on unhealthy food and the government imposes an excise tax on these foods, people respond by reducing, but not eliminating, their consumption of these foods. This reduces their purchasing power. In response, individuals reduce their consumption of relatively expensive fruits and vegetables and other healthy foods, and consequently, this leads to little change in weight.

The successful public health strategy of using tobacco taxation to reduce smoking presents a strong case for considering an economic approach in the context of obesity. Tobacco taxation has been recognized internationally as one of the most effective population-based strategies for decreasing smoking prevalence and consumption and the adverse health consequences [6]. Historically, the effectiveness of taxation as a tobacco control measure has been evaluated in the context of price elasticity of demand, the extent to which the consumption of a product (cigarettes) falls or rises after a change in its price. Recent research estimates that, in high income countries, a 10% increase in cigarette prices results in a 3% to 5% decrease in demand for cigarettes among adults [7]. While price elasticity estimates are comparable among high income countries [8], the impact of taxation appears to be greater in low and middle income countries, where smoking rates are generally higher and tobacco control policies weaker [9].

Existing reviews examining the effects of economic incentives or disincentives on food consumption, physical activity and/or obesity, including a recent brief Canadian parliamentary report [10], conclude that little is known about the impact of various economic instruments on healthy eating and physical activity or on their effectiveness in preventing and controlling obesity in general, or that the impact will be modest [11-14]. However, despite an incomplete evidence base, policy makers still need to select and implement interventions that may have a significant population health impact. Given that the current situation is described as 'a cacophony of policy in which different analyses and policy solutions have been developed and proffered, each clamouring for support, funding and adoption'[15], there is clearly an urgent need for clear and solid evidence to emerge and be synthesized to guide policy decisions.

The purpose of this project was to synthesize existing evidence regarding the impact of economic policies targeting obesity and its causal behaviours (diet, physical activity), and to make specific recommendations for the Canadian context. To achieve this, we adopted Arksey and O'Malley's [16] methodological framework for conducting scoping reviews. Scoping reviews are distinct from systematic reviews in that a) they often address broad topics where a variety of study designs and secondary topics may be relevant, b) they are less likely to formally assess the quality of included studies or use study quality criteria to guide the synthesis of data, and c) they are used to identify parameters around a body of literature, and to identify gaps in the existing body of research. This study's scoping review consisted of two main phases: 1) a structured literature search and review, and 2) consultation with experts in the research field through a Delphi survey and workshop. The former phase focused on selecting and reviewing empirical studies that look at weight outcome and use population level survey data. Given that our interest is in the application of population-level intervention such as taxes and subsidies and given the concern about the substitution effects (discussed earlier), we believe that the evidence from studies of this type are the most relevant. Our study selection thus distinguishes our review from recent reviews such as that of Thow and colleagues [17] which covered both empirical and simulation studies that estimated the effects of subsidies and taxes on specific food products on consumption, body weight and chronic conditions. As a point of comparison, Thow et al. [17] included a total of 24 studies but only 6 of these investigated body weight using population survey data, while our review assesses 20 studies of this type. The latter expert consultation phase was a critical component of the review. As the study of economic interventions for obesity is a relatively nascent area of inquiry, much of the knowledge may not appear in the published or grey literature. Soliciting input from experts regarding an incomplete body of evidence was deemed a necessary step.

### Structured Literature Search and Review

The search strategy was designed in consultation with an information coordinator from the Ontario Tobacco Research Unit (OTRU). Detailed and extensive searches were conducted on Medline, PsycInfo, PubMed, Econlit, Policyfile, Pais International, OVID, Web of Science, Cochrane Reviews and Google Scholar from September to December 2009. Searches were also performed on a range of grey literature sources including NBER, U.S. Department of Agriculture (USDA)'s Economic Research Service, AgEcon Search, and other governmental agency websites. Search term combinations were used to identify relevant studies in the nutrition domain: 'overweight, fat, diet, nutrition, caloric, weight, obesity, BMI, consumption, demand, intake' with 'taxes, subsidy, intervention, economic policy, transfer program, income support, WIC, food stamp, cash transfer, agriculture subsidies, farm policy'. Reviews were done of retrieved primary and review article reference lists (including those from previously published systematic reviews); hand searches of key nutrition and health economics journals (to December, 2009), and; expert panel members were asked to review the final reference list for completeness. In May 2010, an updated literature search was completed that focused from December 2009 to May 2010.

**Figure 1:**
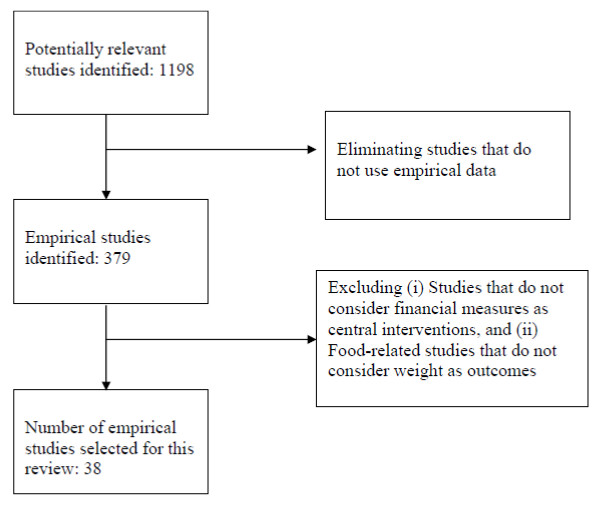
**Selection of empirical studies**.

This comprehensive search resulted in 1198 potentially relevant studies. The initial screening identified 379 studies that employed empirical analysis. Next, studies were selected that focused on financial measures such as prices, subsidies, taxes or income transfer programs as the central intervention. Given the availability of an existing comprehensive review [18] and limited time, we excluded studies that focused on the effects of food prices on food consumption and demand. Instead, studies that explicitly focused on weight outcomes (such as obesity and body mass index), physical activity or caloric intake were assessed. Finally, the review focused solely on observational or randomized controlled trial (RCT) studies that estimated behavioural responses and hence, excluded simulation studies. These requirements further reduced the number of studies reviewed to 38 (see Figure 1) which included 20 studies assessing tax or food subsidies, 4 studies assessing agricultural policies and subsidies, 4 studies assessing physical activity outcomes (tax credits, gas prices, road congestion taxes), and 10 studies assessing targeted income transfer programs. In addition to these empirical studies, we identified 7 relevant reviews. Data from each empirical study was abstracted (e.g., authors, study location, year of publication) including some analytical detail such as design and key findings. Next, the abstracted information was collated and summarized in chart form.

### Expert Panel

To supplement the literature review, an expert panel was convened to contribute input to the literature search strategy, identification of grey literature, and then to assess the strengths and limitations of different economic approaches with a view to proposing specific recommendations for the Canadian context. Expert consultation is an increasingly acceptable source for gathering evidence about a topic particularly when the extant literature is weak [19]. Panel members were identified through an initial literature search for researchers prominent in the field of economics and obesity. Each panel member was also asked to nominate others who should be on the panel. We sought to recruit a varied panel of experts who had published in the area of economic instruments and obesity and were familiar with the literature concerning a broad range of economic instruments. The primary inclusion criteria included publications on economic interventions or consequences of obesity as indicative of specialist knowledge. Turoff [20] recommends panels between 1 and 50. Based on project time lines and cost considerations, we recruited 12 experts from Europe (n = 2), Canada (n = 3) and the United States (n = 7).

We adopted a Delphi survey approach in consulting with the experts. The Delphi survey is a mixed-method research approach, designed for exploring the range of opinions, and exploring (or achieving) consensus on a specific topic. The technique is considered particularly useful in areas of limited research or in areas where there is controversy, debate or lack of clarity [21]. The Delphi has been successfully applied to a range of issues, including views on the most suitable monetary incentives on food to stimulate healthy eating [22]. A four-round conference style format was applied in the current project to examine economic instruments for addressing obesity. During round one, telephone calls were conducted with all panel members to identify potential economic instruments and to confirm the project's literature search strategy. Based on these discussions and the reviewed literature, a survey was created that listed the most commonly reported economic instruments (see Table 1). In round two, this survey was sent to all participants with the request to rate each instrument in terms of its potential impact on obesity, consumption, its cost-effectiveness, potential for unintended benefits or harm, equitability, and political feasibility (see [2]). Experts were then asked to return their responses to a nominated facilitator external to the Delphi process. Respondent names were removed, replaced with a number and then forwarded to the first author. During the third round, questionnaires were returned to each individual expert, containing a summary of their score for each item, along with the score for the group as a whole. Panel members were invited to review their individual ratings against these group means, and resubmit their responses with changed or unchanged scores. Final responses were returned to the facilitator and forwarded to the lead expert. Group means were calculated for each item and then ranked according to their score within each major type of economic instrument. This ranking represented the group's consensus, and was distributed via email to the expert panel with summary statistics purposely timed to precede a 1.5 day long in-person panel meeting held in Toronto. At this meeting, panel member opinions and views on their Delphi rankings and attendant recommended policies were solicited.

**Table 1:** Delphi Survey Results (N = 12)

	Impact on consumption		Impact on PA		Impact on obesity		Cost-effective		Unintended benefit		Unintended harm		Equitable		Politically feasible	
**Intervention**	**Mean**	**IQD**	**Mean**	**IQD**	**Mean**	**IQD**	**Mean**	**IQD**	**Mean**	**IQD**	**Mean**	**IQD**	**Mean**	**IQD**	**Mean**	**IQD**

Beverage tax	2.9	0			2.1	0	2.9	0.5	2.5	1	2.4	1	2.3	1	2.5	1

Food tax	2.7	1			2.2	0.5	2.8	0.5	2.4	1	2.8	1	1.9	0	2.1	0

Fruit & Veg subsidies	2.9	0			2.1	0	2.3	1	2.9	0	2.1	1.5	2.5	1	2.5	1

Child fitness tax credit			2.3	1	2.1	0.5	2.1	0.5	2.8	0.5	2.0	0	2.1	1	3.2	1

Adult fitness tax credit			2.3	1	1.8	0	2.0	0	2.5	1	2.0	0	2.0	0.5	2.9	0.5

Public transit tax credit			2.1	0	2.0	0	2.2	0	3.1	0.5	1.9	0.5	2.7	1	3.2	1

Sporting equipment tax credit			1.9	0	1.7	1	1.7	1	2.3	0.5	1.9	0	2.1	0	2.6	1

Subsidised PA programs			2.5	1	2.0	0	2.1	0	2.6	1	2.1	0	2.3	0	2.5	1

Road congestion tax			2.1	0	1.7	1	2.5	1	3.4	1	1.9	0	2.4	1	2.3	1

Income transfer unrestricted	2.0	1.5	1.7	1	1.9	1	1.8	1.5	2.9	0.5	2.7	1	2.0	1.5	2.5	1

Income transfer healthy food	2.9	0.5			2.1	0	2.6	1	2.8	0.5	2.3	0.5	2.4	1	2.8	0.5

Income transfer PA			2.3	1	1.9	0	1.9	0	2.5	1	2.0	0	2.3	1	2.2	1

Agricultural subsidies	2.4	1			2.3	0.5	2.9	0.5	2.9	1.5	2.6	1	2.1	1	1.7	1

Agricultural R&D rebalance	2.4	1			2.2	0.5	2.5	1	2.9	0.5	2.3	1	2.8	0.5	2.7	1

Notes: Not at all/None = 1; Low = 2; Moderate = 3; High/A Lot = 4.

PA = Physical Activity; IQD = Inter Quartile Deviation; indicates the distance between the 25th and the 75th percentiles. A smaller IQD represents greater consensus.

## Results

### Delphi Survey Results

Table 1 presents the findings of the final Delphi survey completed before the panel meeting. First, all economic instruments were rated as having a relatively modest impact, if any, on obesity. Economic instruments targeting consumption were rated higher than those targeting physical activity. Of the instruments, changing agricultural subsidies was rated as having the highest potential impact on obesity but also the lowest in terms of feasibility. Food taxes were rated second highest in terms of obesity impact but also scored highest in terms of potential for unintended harm and being inequitable. Fruit and vegetable subsidies and beverage taxes were rated similarly in terms of potential impact on consumption and obesity but differed in terms of potential for unintended benefit and cost-effectiveness.

The panel meeting discussions largely were in line with these survey findings and concentrated on three broad considerations: 1) reviewing agricultural policy and subsidies; 2) implementing a tax on caloric sweetened beverages; and 3) examining how fruit and vegetable subsidies can be targeted. Attention now turns to discussing each of these after a brief overview of the research evidence. The consideration of food taxes, income transfers, and economic instruments for promoting physical activity is briefly noted.

### Reviewing Agricultural Policy and Subsidies

#### Evidence

Several authors and commentators [23-26] have taken the strong correlation between increased farm subsidies and the rise in obesity rates in the US since the 1970s as evidence that they are causally related. They argue that these subsidies have reduced the prices of soybeans, corn and other farm commodities. These subsidized commodities - which are cheap sources of sugar and fat for processed foods - have lowered the price of processed foods. The lower prices of these processed foods in turn have contributed to their over-consumption, which has contributed to the increase in obesity. Additionally, subsidies can provide incentives for technological innovations which have increased the availability of ingredients such as corn-based sweeteners.

Several empirical studies have challenged the claim that farm subsidies have increased rates of obesity. Miller and Coble [27] investigated whether farm subsidies make retail food products in the US more affordable using annual time series data from Economic Research Service of USDA for the period 1961-2002. The affordability of food, their outcome variable, is captured by the proportion of disposable income spent on food while farm subsidies are measured by direct payments to farmers. In addition to farm-to-retail price spread and consumer income, their model also includes agriculture's total factor productivity (TFP) to shed light on the effect of changes in technology on food affordability. They estimated this regression in the aggregate as well as across 6 specific food groups. The results indicated that direct payment impact on food affordability was not statistically significant. In contrast, the positive and statistically significant TFP implies that advances in agriculture technology have increased the affordability of foods. Furthermore, these results are consistent across food groups. These findings [27] provide empirical evidence that cheap food prices are mainly caused by increases in agricultural efficiency, perhaps enhanced by public R&D subsidies, over the last several decades rather than by farm subsidies.

Beghin and Jensen [28] used historical data to examine whether US farm policies for sweetener crops have affected the consumption and composition of sweeteners in the US diet. The data showed that commodity programs have raised the price of cane sugar and decreased the price of corn. At the same time, agricultural R&D expenditure lowered the cost of corn more than that of sugar. Thus high fructose corn syrup became an inexpensive substitute for sugar in food and beverages. However, they emphasize that the effect of policy on ingredient prices has become less important over time, with the current farm value share in sweetened food being below 5%. They also noted that increased consumption of sweetened foods and beverages are observed in other countries which have different or no commodity programs.

Alston and colleagues [29] examined US and international data to shed light on the impact of farm subsidies on commodity prices and of commodity prices on food retail prices. They found that farm subsidies have had very modest (and mixed) effects on the total availability and prices of farm commodities that are the most important ingredients in more-fattening foods. Second, such small commodity price impacts would imply very small effects on costs of food at retail, which, even if fully passed on to consumers, would mean very small changes in prices faced by consumers.

Additional evidence suggests that agricultural R&D subsidies contribute to high productivity and thus reduce the prices of commodities. Alston et al. [30] studied the contributions of US public agricultural research and extension investments over 1890-2002 to state-specific agricultural productivity for the period 1949-2002. They found that both state and federal agricultural R&D investment yielded high returns. Specifically, a one dollar increment in investments in agricultural research and extension by 48 U.S. states generated own-state benefits of between $2 and $58 and averaged $21 across the states. They suggested that the returns would be even higher if the spill-over effects across the states were taken into account (between $10 and $70 per research dollar across the states, with an average of $32).

##### Panel Conclusions

Panelists agreed that agricultural support policies were influential in the rise in rates of overweight and obesity. However, panelists conceded that there is no strong empirical evidence linking agricultural support policies to the growth in obesity. Nevertheless, panelists felt strongly that the lack of evidence was a result of the complex causal pathway and considerable time lags between policy changes and resulting changes in population levels of obesity. While panel members felt that modifying agricultural policies would have the biggest impact on reducing obesity, such modification also scored the lowest for feasibility. Limiting or eliminating farm subsidies to commodity farmers is unlikely to rapidly change a complex agricultural system that has evolved over decades. More attention could be directed toward the impact of agricultural R&D supports which may have over time lowered cost of added sugars and fats derived from corn, soy, potatoes and other farm commodities. In turn, these added sugars and fats have found their way into much processed food. It was also suggested that subsidies may have an impact on food formulation rather than just price alone. Holding consumer food price equal, a subsidy that (for example) increases the affordability of sweeteners could lead to undesirable dietary outcomes without affecting product price.

##### Agricultural subsidy-specific recommendations included

a) Create and implement an effective health filter to review new and current agricultural polices to reduce the likelihood that such policies have a deleterious impact on population rates of obesity. Specifically, an agricultural support policy should become secondary to a food and health policy.

b) Restructure R&D investment and subsidies to promote increased development of fruit and vegetable production and distribution. Measures to raise domestic supply of fruits and vegetables can also be complemented by lowering tariffs on imported fruits and vegetables.

c) Develop transportation and subsidized revenue insurance policies to assist farmers who grow fruits and vegetables, widely considered a riskier crop than other agricultural products. Farmers should be engaged as 'anti-obesity' partners.

d) Develop measures that promote easy access to fruit and vegetables for Canadian households. For example, both the European Union and the United States have recently implemented policies to actively promote farmers' markets.

### Caloric Sweetened Beverage Tax

#### Evidence

We identified 5 recent empirical studies that evaluated the effect of beverage taxes currently adopted in a number of US states on body mass index (BMI) and obesity [31-35]. The focus of these studies on the effect of beverage taxes on weight outcomes differs from that of the beverage price elasticity studies, such as those reviewed in [18], which estimated the effects of beverage prices on beverage consumption.

All of these 5 studies used cross-sectional data. Two studies examined the effect of soda taxes on adult weight. Kim and Kawachi [31] investigated the effect of these taxes on state-level obesity prevalence, using state-level taxes on soft drinks and snacks and the 1991-1998 cross-sections of data from the U.S. Behavioral Risk Factor Surveillance System (BRFSS). They found weak statistical evidence (*p*-value = 0.09) that states that had repealed a soft-drink or snack-food tax were 13 times more likely than states with a tax to experience a relative increase in obesity prevalence. In addition, states without a soft drink or snack food tax were four times more likely (albeit statistically insignificant p-value = 0.25) than states with a tax to exhibit a high relative increase in obesity prevalence.

Fletcher et al. [32] also used the BRFSS data, but for the period 1990-2006, and focused on the effect of soft drink taxes on individual weight outcomes. Using their preferred model, they found that soft drink taxes influence individuals' BMI but the impact was small in magnitude. The authors also reported that tax effects on weight outcomes were larger among low income groups. This result suggests that such taxes may not be regressive as is commonly assumed.

Powell and colleagues [33] examined the effect of soft drink taxes on adolescent weight using Monitoring the Future data for the period 1997-2006. They found no statistically significant relationship between soda taxes and adolescent weight outcomes but did find a weak economic and statistically significant relationship between the vending machine soda tax rate and BMI among adolescents at risk for overweight.

Fletcher et al. [34] considered the effect of soda taxes on children and adolescent weight using the U.S. National Health and Nutrition Examination Survey (NHANES). In addition to examining weight, they assessed whether higher soda taxes lead to increased consumption of milk and juice. Their econometric framework controls for unobserved state-level characteristics (through the inclusion of state-specific fixed effects) that could be correlated with soft drink taxes (failure to control for unobserved characteristics could lead to misleading estimates). They found that soft drink taxes lead to a modest reduction in soda consumption by children and adolescents, but have no effect on children and adolescents' net weight due to an increase in consumption of whole milk (but not juice or juice-related drinks). They concluded soda taxes, as currently practiced, do not reduce weight in children and adolescents. However, as children and adolescents appear to substitute whole milk for soft drinks in response to soda taxes, there may be unexplored broader nutrient benefits of soda taxes for children and adolescents.

Finally, Sturm et al. [35] investigated the effect of state sales taxes for soft drinks on children's consumption of soft drinks and weight gain. They used the tax rates that were in effect in January 2004 and matched them to the fifth-grade wave of the Early Childhood Longitudinal Study individual-level data. Their results indicated that existing taxes on soft drinks do not substantially affect overall levels of soda consumption or obesity rates. However, they found that subgroups of at-risk children (i.e. children who are already overweight, come from low-income families, or are African American) may be more sensitive than others to soft drink taxes, especially when soft drinks are available at schools. They suggested that a larger soft-drink tax is required to generate meaningful changes in consumption and weight outcomes.

#### Panel Conclusions

Three-quarters of the panel recommended moving forward with a tax on caloric sweetened beverages; these include soda, energy drinks, sports beverages and many juices and iced teas that are sweetened with sugar, corn syrup, or other caloric sweeteners. Sugar-free diet drinks, diet beverages, sugar free juice, and flavoured milk would be tax exempt. There was agreement that while such a tax may in itself have a modest impact on obesity, it could be quite powerful in its impact over time, and have a synergistic effect with other tax, legislative, and educational initiatives to address obesity - this may be largely in relation to changing norms about dietary consumption.

In some respects, such a recommendation was described as a "leap of faith" given the incomplete evidence base. Nevertheless, most panelists felt that a tax on caloric sweetened beverages is justified, for several reasons. First, it was noted that unlike fast foods, caloric sweetened beverages "served no nutritional value". Second, there was no indication from the empirical evidence that such a tax would be regressive and unfairly penalize low income individuals and households. Finally, just as the (rather successful) tobacco control policies were introduced with imperfect information concerning their effectiveness, so too, many obesity control policies will need to be introduced in the context of imperfect evidence. The actual impact of public policies will only be clear once they take effect.

The uncertainty surrounding the impact of such taxes is likely due to the low level of existing tax rates since the current caloric-sweetened beverage taxes were not designed and implemented to address obesity. These taxes are too low to generate a meaningful influence on caloric consumption and body weight, and lack sufficient variability across jurisdictions and time to generate precise causal effect estimates. Even so, there is evidence that low income individuals and children are the most sensitive to changes in food prices generally. Additionally, there is strong empirical evidence that the consumption of soft drinks is responsive to its prices (for every 10% rise in prices, consumption declines 8 to 10%; see [18]). Therefore, it is reasonable to expect that a sufficiently high tax imposed on caloric sweetened beverages would be likely to reduce consumption. However, experts acknowledged that such data relies on many assumptions that may not hold during implementation. Unknown effects regarding substitution, compensatory behaviour, and producer response all serve to lower confidence in such claims.

In terms of magnitude, small taxes on soft drinks will likely do little to lessen soft drink consumption or prevent childhood obesity. The panel experts noted that the US tax rates up to 7.25% at the time did not have the desired effect on weight outcomes. The level of the taxes will depend on where the soft drinks are sold (vending machine, convenience stores, and supermarkets) but experts suggest a minimum tax of around 20% of the price. For example, New York State was considering an 18% tax in 2009. Further, taxes should be calculated and implemented on a unit basis, rather than percent of price to avoid quantity discounts. Panellists also suggested that the taxes be salient. That is, taxes are likely to have larger impacts if they are made visible to consumers [36].

The proposed soft drink tax would also deliver several other benefits. First, the revenue from this tax could be used to fund other initiatives to reduce obesity, such as the introduction of free water fountains at public places. Tax revenue could also be used to fund fruit and vegetable subsidy programmes. Second, a decline in soft drink consumption will decrease sugar intake, which may provide health benefits beyond the direct effects on obesity (see [37] for the list of cardiovascular diseases associated with sugar consumption). Third, experts noted that fast foods and soft drinks are often consumed together, so they may be complementary goods. Thus, higher soft drink prices may reduce the consumption of fast foods.

Three panel members did not believe such a tax would have an impact on obesity prevalence, claiming that consumers would substitute unhealthy foods not subject to the tax if faced with higher prices for caloric sweetened beverages. They favoured addressing the root economic forces at play, which they viewed as the agricultural R&D and commodity subsidy policies that have lowered the prices and increased the consumption of energy dense foods. An alternative strategy was taxing sugar as an input to caloric sweetened drinks rather than consumer-facing taxes. This might encourage health-promoting reformulations as well as put some upward pressure on consumer prices on such beverages.

##### Specific recommendations for the Canadian context included

a) Apply the tax on the amount of caloric sweetener in the beverage (e.g., 10 cents per ounce of sweetener);

b) Rationalize the tax in terms of broader health benefits as opposed to a single focus on obesity. Decreasing sugar consumption has health benefits beyond those associated with lowering obesity;

c) Combine the implementation of such a tax with targeted unsweetened beverages and/or fruit and vegetable subsidies, or in other obesity prevention interventions, and;

d) Monitor any unintended consequences of the tax implementation in terms of producers' formulation responses.

### Food taxes

Another policy option is to tax certain foods linked to obesity. The panel viewed the evidential base for policy in this area to be more compelling than the evidence to support a beverage tax. In particular, they were swayed by the 4 longitudinal studies that suggest that low fast-food prices increase weight outcomes [38-41] and 6 studies employing cross-sectional data [42-47]. However, panel members did not recommend proposing such taxes at this time despite the empirical evidence. There are a number of difficulties with the design and implementation of food taxes that require further research before specific recommendations can be made.

The greatest challenge with food taxes is defining the scope of foods to be taxed that fall under the category of energy dense, unhealthy foods. Complex decision rules to capture all foods is likely to be unfeasible and involve large administrative costs which might even exceed the revenue from taxing such foods. A more narrow scope may not achieve the goal because consumers will be able to substitute from one energy-dense food that is taxed to some other energy dense food that is not taxed. This could be averted by defining a tax on food types as proportional to its content of sugar for example. However, deciding on what is actually being taxed is not without controversy.

Another important concern with food taxes is the issue of food insecurity. For low income individuals, cheap, high energy foods may be the primary source of energy. Accordingly, these individuals may use a higher share of their income to pay food taxes than their wealthier counterparts. In other words, food taxes are more likely to be regressive. An additional challenge is that some foods have a mixture of good and bad nutrients, such as cereals with added sugar. Taxing these foods might eliminate both good and bad sources of nutrients which in turn might have a deleterious impact on health.

### Fruit and Vegetable Subsidies

#### Evidence

We did not identify any study evaluating the impact of fruit and vegetable subsidies on overweight and obesity directly. However, there is evidence on the effect of prices of fruits and vegetables on weight outcomes, including 4 longitudinal studies [38-40,48] and 3 cross-sectional studies [42,43,46]. Sturm and Datar [38] used longitudinal data on children followed from kindergarten through third grade in the U.S. Early Childhood Longitudinal Study. They found that changes in children's weight were positively related to the price of fruits and vegetables but not to changes in meat, dairy, or fast-food prices. Specifically, an increase in the price of fruits and vegetables by one standard deviation raised children's BMI by 0.11 units by third grade (equivalent to a BMI price elasticity of approximately 0.05) based on analyses by Powell and Chaloupka [13]. Their subpopulation analysis suggest that children living in poverty and those at risk for overweight were roughly 50 and 39 percent, respectively, more price sensitive compared with their non-poor and not-at-risk counterparts.

Sturm and Datar [48] followed up their 2005 study by expanding the panel data to include the fifth grade students. They found that one standard deviation increase in the price of fruits and vegetables increased children's BMI by 0.09 units by third grade and by 0.18 units by fifth grade. This result confirmed their previous finding that children's BMI responds to changes in fruit and vegetable prices. More importantly, their results suggest a consistent long-term effect of fruit and vegetable prices on children's weight outcomes.

Sturm and Datar's [38,48] findings are consistent with findings reported by Powell and Bao [40]. The latter study used panel data from the 1979 cohort of the National Longitudinal Survey of Youth and price data for fruit and vegetable and fast food price from the ACCRA (American Chamber of Commerce Research Association). They found that a 10% increase in the price of fruits and vegetables was associated with a 0.7% increase in child BMI.

Another important piece of evidence concerning the effect of fruit and vegetable prices on child weight is from Powell and Chaloupka [39]. Using panel data from the Child Development Supplement of the Panel Study of Income Dynamics, their fixed effects model showed that higher fruit and vegetable prices are significantly related to a higher BMI percentile ranking among children, with greater effects among low-income children: fruit and vegetable price elasticity for BMI was estimated to be 0.25 for the full sample and 0.60 among low-income children.

For adolescents, there is weaker evidence that fruit and vegetable prices have an impact on body weight [42]. However, one study has found adolescents' weight to be sensitive to the price of fruits and vegetables [43]. In particular, for both males and females, the effects of the prices of fruits and vegetables (and fast-food meals) at the 90th or 95th quantiles were found to be relatively large, between three to five times greater than across the distribution as a whole. Based on this result, the authors suggest that subsidies for fruits and vegetables would have the greatest effect on reducing the weight of adolescents most at risk for overweight. Finally, Beydoun, Powell and Wang [46] report a positive correlation between fruit and vegetable prices and adults' BMI.

#### Panel Conclusions

In contrast to concerns about food taxes, panel members were uniformly in favour of fruit and vegetable subsidies - primarily targeting children and low income households. It was believed that the evidence clearly demonstrated a link between lower obesity risk and greater fruit and vegetable consumption although the mechanisms for this relationship are unclear. Subsidies can become largely an income transfer when people already consume some amount of the goods that are targeted by the subsidy. In such a case, there is a risk that the additional income is used for other goods including energy dense foods which counters the goal of the subsidy. However, this is unlikely for fruits and vegetables which are under-consumed by the population on average and in particular, by those with lower income (see [49]). This explains the recommendation proposed here that fruit and vegetable subsidies be targeted at low income people and children only. For children, encouraging them to eat fruit and vegetables will likely reduce the childhood obesity problem and may help them to develop a healthy habit of consuming fruit and vegetables in later years.

In terms of the subsidy coverage, both fresh and frozen as well as canned fruit and vegetables should be eligible for subsidy. For low-income adults, one way to deliver these subsidies is through grocery cards or debit cards. These cards could be potentially connected with the Canada Revenue Agency databases for monitoring and reimbursement purposes. For children, free fruit and vegetables could be offered at schools. One example is the School Fruit Scheme implemented in the European Union http://ec.europa.eu/agriculture/markets/fruitveg/sfs/index_en.htm.

It was also noted that the total costs of a diet include both the monetary cost of buying the ingredients and the time cost of preparing the ingredients for consumption. Unlike soft drink or junk foods, fruit and vegetable preparation takes greater time. Consequently, manipulating only the prices of fruit and vegetables may not be enough to generate behaviour changes because the time cost of preparation may still result in people failing to consume them (even if they buy them). Therefore, subsidy measures should be accompanied by measures to promote convenient cooking of these increased fruits and vegetables. In this regard, ready-to-eat fruit and vegetables provided at school meals are more attractive than fruit and vegetable subsidies targeted at low income people.

Finally, targeted fruit and vegetable subsidies will generate higher demand and may increase fruit and vegetable prices for non-subsidized groups. This might be more relevant in Canada where fruit and vegetable supply is not perfectly price elastic. For example, the cost of fruit and vegetables in Canada increases in the winter time. The extent of the price increase depends on the incremental costs to farmers and distributors of increasing production. The higher prices will likely put pressure on the government budget. This issue highlights the need to see the synergistic effects of economic interventions. Fruit and vegetable subsidies may need to be supported by any revenue generated by beverage taxes, while agricultural subsidies will need to be shifted to support fruit and vegetable production.

##### Specific recommendations for the Canadian context included

a) Implement subsidy coverage and ensure that eligible products include both fresh and frozen as well as canned fruit and vegetables. For low-income adults, such subsidies may be delivered through grocery or debit cards. These cards can be connected with the Canada Revenue Agency for monitoring and reimbursement purposes;

b) Offer children and youth free fruit and vegetables at school;

c) Dedicate portions of beverage tax revenue to fund fruits and vegetable subsidies, and;

d) Shift agricultural policy and subsidies to enhance the production and distribution of fruits and vegetables.

### Physical Activity

#### Panel Conclusions

There was less confidence among the panel members that economic instruments as defined within the scope of their discussions (i.e., taxes and/or subsidies) was an effective means to increase physical activity at the population level. In terms of economic instruments, it was proposed that such instruments might be more effective targeting consumption as opposed to energy expenditure. Additionally, while panel members recognized the two-sided nature of issues related to physical activity - i.e., economic measures to increase physical activity and economic measures to decrease sedentary behaviour, suggestions for economic measures that penalized inactivity were considered to be unrealistic. However, such broad conclusions need to be considered in the light of very little evidence concerning the impact of economic measures to increase physical activity participation.

Tax credits were seen as rather ineffective at encouraging physical activity amongst the sedentary; indeed they were deemed to provide windfall gains to those who already participate in physical activity programs and hence were inequitable. Panellists suggested that money would be better spent on subsidizing physical activity programs, particularly those designed for children and low income groups. However, there was a concern that this might be directed at 'organized sports' which does not necessarily equate to increased physical activity. In general, there was greater support for examining how subsidies might be targeted at specific populations to increase physical activity participation - e.g., immigrant populations, single mothers, etc. There was some speculation that gas taxes are one way to shift modes of transportation but revenue would need to be directed to developing physical activity facilities.

In summary, the panel suggested that there was insufficient evidence to clearly recommend specific tax credits or subsidies to promote physical activity. This is not to discount any policy changes in this area. At the least, public funds should be transferred from potentially inefficient economic measures to encourage increases in physical activity (e.g., the Children's Fitness Tax Credit; see [50]) to economic measures that show more promise (e.g., subsidized participation for targeted populations). The promise of such economic measures should be tested in a matching program of research to determine the actual effects of such measures on increasing physical activity participation and reducing obesity.

### Income Transfers

#### Panel Conclusions

In addition to using taxes and subsidies to alter the relative prices of healthy foods versus unhealthy foods, another potential tool to address obesity is to use income transfer programs. This project's review of empirical research distinguished two types of transfer programs. The first type generally involves income support whose main goal is to address poverty and is referred to as 'unrestricted' income transfers. There are many such programs in the US, including for instance, Temporary Assistance for Needy Families (TANF) for single mothers, Disability Insurance, and Supplemental Social Security Income for older adults.

Another type of transfer programs - 'restricted' income transfers - can be redeemed for food and beverages only. One example is the Supplemental Nutrition Assistance Program (SNAP) (formerly called the Food Stamp Program). Participants in the SNAP program are distributed debit cards (historically, paper denominational stamps or coupons worth $1, $5, and $10) that can be used to purchase any food or food product intended for human consumption, except alcoholic beverages, tobacco, and hot meals and hot food products prepared for immediate consumption. We did not identify a transfer program that is targeted directly at promoting physical activity.

Income transfer approaches did not receive any support from the panel members in terms of addressing obesity. This was primarily due to the perception that existing evidence did not clearly support a simple inverse relationship between income and obesity. That is, obesity impacted all socioeconomic strata. The panel felt that there are many good reasons to consider income transfers but obesity prevention was not one of them. Overall, the panel felt it was a safer course of action to focus on targeted subsidies although it was appreciated that there exists a nuanced distinction between income transfers and subsidies.

## Discussion

A comprehensive combination of educational, regulatory, direct provision, and economic policies will be essential for effectively tackling the public health burden of obesity. Economic interventions by themselves are not the answer but should be one component of such a comprehensive approach. In terms of economic interventions, shifting from empirical evidence to policy recommendations remains challenging. Current evidence is not sufficiently strong to provide clear policy direction. Additionally, the nature of the experiments needed to provide definitive evidence supporting certain policy directions is likely to be complex and potentially infeasible. However, these are not reasons to take no action. Engaging research experts for their informed opinion is an important source of evidence particularly when the evidence base is incomplete [19]. The process reported here was informative for focusing attention on broad strategies for policy consideration and greater research attention. It should be acknowledged, however, that generalizations are limited in using the Delphi method - another panel may reach different conclusions for example [21]. As research increases in this area, broadening the size and scope of expertise contributing to such panels will be informative in the future.

Panellists agreed that the most important priority was to modify agricultural support policies and food subsidies so as to both lower the prices, and increase the availability, of fruit and vegetables. US-based experts were most adamant about the potential deleterious impact of agricultural policy on obesity prevalence in North America. In Canada, there has been little empirical research on the effect of agricultural policies on food choices and obesity outcomes. It seems plausible, however, that Canadian agricultural support policies have, at best, only a modest effect on obesity. Canada has little influence on prices of obesity-linked commodities such as soybeans and corns (Canada is a large agricultural exporter, but it is a small player in most commodity markets, except for wheat and canola oil). Also, the size of agricultural subsidies in Canada is relatively small compared to those in the United States and Europe. Despite this lack of research evidence there are still other agricultural policies that are likely to have had an impact on food choices and contributed to rising obesity in Canada. These policies are reviewed and assessed qualitatively by Cash, Goddard, and Lerohl [51]. The authors note that Canada's dairy supply management program has encouraged consumption away from fluid milks and towards dairy products that have higher fat and sugar content. For example, milk processors will pay suppliers less for milk used in the manufacture of ice cream than for milk that is processed into fluid milk.

The majority of the panel members recommended the implementation of a tax on caloric sweetened beverages while at the same time subsidising fruits and vegetables for children and low-income households. There is evidence that adult weight is modestly responsive to beverage taxes. For children and adolescents, such taxes lead to only small weight reductions, but they may induce a substitution from soft drinks to whole milk. Adolescents at risk of being overweight may also experience weight reduction. Additionally, there is consistent evidence that lower prices of fruits and vegetables are associated with lower child weight. For adolescents and adults, the evidence also suggests that weight is sensitive to fruit and vegetable prices.

Given that the evidence supports an effect of food prices on weight outcome, the key question is the magnitude of this effect. According to studies that were selected and reviewed, price effects are small. However, it might be inaccurate to conclude that prices have small effects on weight outcomes. The estimated price effects should only be considered as the lower bound of price effect, as there are a number of factors that might cause the effect to be underestimated. First, the current state-level soda and snack taxes in the US may be too small and lack the variation necessary to help identify meaningful effects on people's weight. To date, none of the implemented food taxes were designed with the primary purpose of addressing obesity. For example, average state taxes imposed on soda and soft drinks are very low, at $0.0425 on a $1.00 bottle of soda when sold through grocery stores [33]. This is in contrast with cigarette excise taxes of as much as $2.75 on a pack of cigarettes (in New York) and the combined state and federal taxes that more than double the retail price of cigarettes in many states [52].

Second, prices of fast foods are determined in part by demand conditions, therefore they may not be exogenous. Goldman et al. [53] point out that if food prices are determined by both supply (i.e. manufacturers) and demand conditions, the effect of prices on weight outcomes will be underestimated in the empirical studies. Third, measurement errors in weight and price data might bias price effects downward. The information on weight and height used to calculate BMI are mostly self-reported. At the same time, there are limitations to the price data from ACCRA. These data were collected in larger cities and metropolitan statistical areas and as a result are skewed towards higher income households and will produce considerable measurement error when matched to low-income or rural populations. Further, only a small number of food items are surveyed, so the data are not fully representative across food groups. Also, ACCRA does not always continuously sample the same cities, and hence the data are not fully comparable over time.

Fourth, there is an inherent difficulty in estimating the effect of economic factors on weight outcomes. Because these effects follow a nonlinear accumulation pattern (i.e., as Katan & Ludwig [54] explain, in response to continuous increases in caloric intake, weight does not increase continuously, but rather can adjust in discrete jumps), one needs to distinguish between short term impacts (which can be modest in magnitude) and long term impacts (which as Goldman et al. [53] demonstrated, can be substantial). Specifically, a small, short-term (i.e., week-to-week, month-to-month., or even year-to-year) economic impact may add up to a quite large long-term outcome (e.g., 10 calories per day is 3, 600 calories per year, which over 30 years, ceteris paribus, can add up to > 30 pounds of body weight).

In considering the existing evidence, it is most likely that policies will need to be implemented in the face of an incomplete evidence base - and parallels can be drawn with tobacco control - initial tobacco control interventions were not evidence-based but represented sound judgment at the time [55]. Where the empirical evidence is still not sufficiently strong, perhaps the most important criteria for considering a policy is the potential for harm such a policy might cause, rather than the extent of its impact on obesity. Additionally, even a good policy intervention involves some trade-offs. That is, a good policy may hurt some population segment, but on the whole may benefit society. The concern about the regressive nature of tax measures (that is, taxes may impose a larger burden on the poor than the rich) normally fails to take into account the potentially large health improvements resulting from imposing these taxes. It remains to be seen whether the political will exists in Canada to introduce and evaluate such tax and subsidy measures.

## Competing interests

The authors declare that they have no competing interests.

## Authors' contributions

GF and PG conceived of the project, and contributed to the design and execution of the scoping review and Delphi survey with KA, PD, RF and RM. VHN assisted with literature searching, review and analysis. As members of the expert panel, TA, CA, SC, JC, AD, LD, IJ, JL, DL, LP, BT and FW all contributed significant intellectual content that is presented in this report. All authors read and approved the final manuscript.
